# Permanent neonatal diabetes due to a heterozygous INS mutation

**DOI:** 10.1186/1687-9856-2015-S1-P29

**Published:** 2015-04-28

**Authors:** Kha Chin Long, Johari Mohd Ali, Muhammad Yazid Jalaludin, Fatimah Harun

**Affiliations:** 1 Department of Molecular Medicine, Faculty of Medicine, University of Malaya, Kuala Lumpur, Malaysia; 2 Department of Pediatrics, Faculty of Medicine, University of Malaya, Kuala Lumpur, Malaysia

## 

Permanent Neonatal Diabetes Mellitus (PNDM) is a rare disorder where patient presents with diabetes within the first few months of life without autoantibodies associated with type 1 diabetes. The majority of PNDM cases have INS, ABCC8 or KCNJ11 mutations. We present a PNDM case with INS mutation. The proband is a second child of three siblings without family history of diabetes. She was born at term via emergency lower segment caesarean section with good APGAR score. Her birth weight was 2.0kg (<3^rd^ percentile), length 49cm (50^th^ percentile), and head circumference 34cm (50^th^ percentile). She was discharged well at day 3 of life, but readmitted at day 17 of life with hyperglycaemia, sepsis and severe metabolic acidosis, requiring insulin infusion. Despite clinical improvement and resolving sepsis, she remained hyperglycaemic and hence neonatal diabetes was suspected. Her GAD-65 and ICA-512 antibodies were negative. HbA1c and c-peptide at diagnosis were 7.9% and 38 pmol/L (normal range: 297.9–1324) respectively. She was still hyperglycaemic despite receiving total daily insulin (TDI) of 1.5U/kg/day, but not ketotic. She was discharged after 20 days of hospitalization with insulatard administered three times daily (TDI 1.2U/kg/day). During the first 8 months of diagnosis, her metabolic control was good (HbA1c 6.7 - 7.6%) despite low insulin requirement, as low as 0.4U/kg/day. Her metabolic control deteriorated since then (HbA1c 11.9 - 13.2%), with TDI doses ranging 0.7 - 0.9 U/kg/day. Rapid acting insulin was not used due to episodes of hypoglycaemia and unpredictable eating habit and activity levels during her toddler years. Direct DNA sequencing revealed she is heterozygous for p.A24D INS mutation. This mutation has been reported in the literature and known to disrupt preproinsulin processing. Recent evaluation at 51 month showed, negative anti-islet cell antibodies and c-peptide of <30pmol/L (normal range: 297.9–1324). Currently, her developmental milestone is appropriate for her age. However, her height and weight were both below the 3^rd^ centiles. She was recently started on insulin pump to improve her metabolic control and growth.

Written informed consent was obtained from the patient for publication of this abstract and any accompanying images. A copy of the written consent is available for review by the Editor of this journal.

